# Addition of exogenous sodium palmitate increases the IAPP/insulin mRNA ratio via GPR40 in human EndoC-βH1 cells

**DOI:** 10.1080/03009734.2017.1368745

**Published:** 2017-10-05

**Authors:** Camilla Krizhanovskii, Rikard G. Fred, Marie E. Oskarsson, Gunilla T. Westermark, Nils Welsh

**Affiliations:** a Science for Life Laboratory, Department of Medical Cell Biology, Uppsala University, Uppsala, Sweden;; b Södertälje Hospital, Department of Internal Medicine, Södertälje, Sweden

**Keywords:** Amyloid, fatty acids, insulin, islet amyloid polypeptide (IAPP*)*, palmitate

## Abstract

**Background:**

Enhanced IAPP production may contribute to islet amyloid formation in type 2 diabetes. The objective of this study was to determine the effects of the saturated fatty acid palmitate on IAPP levels in human β-cells.

**Methods:**

EndoC-βH1 cells and human islets were cultured in the presence of sodium palmitate. Effects on IAPP/insulin mRNA expression and secretion were determined using real-time qPCR/ELISA. Pharmacological activators and/or inhibitors and RNAi were used to determine the underlying mechanisms.

**Results:**

We observed that EndoC-βH1 cells exposed to palmitate for 72 h displayed decreased expression of Pdx-1 and MafA and increased expression of thioredoxin-interacting protein (TXNIP), reduced insulin mRNA expression and glucose-induced insulin secretion, as well as increased *IAPP* mRNA expression and secretion. Further, these effects were independent of fatty acid oxidation, but abolished in response to GPR40 inhibition/downregulation. In human islets both a high glucose concentration and palmitate promoted increased IAPP mRNA levels, resulting in an augmented IAPP/insulin mRNA ratio. This was paralleled by elevated IAPP/insulin protein secretion and content ratios.

**Conclusions:**

Addition of exogenous palmitate to human β-cells increased the IAPP/insulin expression ratio, an effect contributed to by activation of GPR40. These findings may be pertinent to our understanding of the islet amyloid formation process.

## Introduction

Type 2 diabetes mellitus (T2DM) is characterized by hyperglycemia resulting from impaired insulin production and insulin resistance in peripheral tissues, and is typically diagnosed when β-cell function is reduced by about 50% (1,2). Aggregates of insoluble amyloid fibrils in pancreatic islets is a pathological characteristic in T2DM, and probably of great importance for the development of β-cell failure and impaired insulin production (3,4). The amyloid fibrils consist of islet amyloid polypeptide (IAPP), a 37-amino acid peptide synthesized primarily in β-cells. IAPP is co-localized (5,6) and co-secreted with insulin (7). The IAPP/insulin ratio is normally low, with IAPP levels approximately 1% of insulin levels (8–12). IAPP exerts its effects through binding to the calcitonin receptor in complex with either the receptor activity modifying protein-1 (RAMP1) or RAMP3 (13).

Overexpression and enhanced secretion of IAPP may contribute to pancreatic amyloid formation and development of T2DM. Recent results from transplantation studies indicate that IAPP fibrils or oligomers play an important role in progressive β-cell failure in transplants, indirectly suggesting a similar mechanism in T2DM (14). Obesity and hyperlipidemia increase the risk of developing T2DM. Interestingly, mice fed a high-fat diet (HFD) are characterized by increased plasma IAPP levels (15,16), and in human IAPP transgenic mice islet amyloid appears after a long-term HFD intake (17). In this context it may seem contradictory that in T2DM not only insulin but also IAPP secretion is impaired (10,11). However, *in vitro* studies indicate that insulin prevents IAPP aggregation (18), and it may be that a change in IAPP/insulin ratio, rather than an increase of IAPP per se, is important for amyloid formation. Amyloidogenic forms of IAPP have been shown to trigger Nlrp3 inflammasome activation (19), activating caspase-1-mediated cleavage of pro-IL-1β into mature IL-1β (20). Further, monocyte-derived macrophages from diabetic patients display significantly elevated cleaved caspase-1 and release of IL-1β following treatment with IAPP (21). Thus, it is possible that amyloid deposits, promoted by an increased IAPP/insulin ratio, initiate the islet inflammatory reactions observed, which may further deteriorate β-cell function.

Despite the possible role of IAPP in β-cell failure and T2DM, the effects of fatty acids on β-cell IAPP expression and release are far from well characterized. In addition, *in vitro* studies previously undertaken have in many cases utilized rodent β-cells/islets. In the present study, we employed a homogenous population of human β-cells with the aim to investigate effects and the underlying mechanisms of fatty acids on IAPP and insulin expression and secretion from insulin-producing β-cells.

## Materials and methods

### Cell culture and in vitro exposure

Human EndoC-βH1 cells were cultured as previously described (22). Mouse insulinoma (MIN6) cells were cultured in 25 mmol/L glucose DMEM supplemented with 15% FBS. Palmitate (sodium salt, Sigma-Aldrich) exposure media were supplemented with 2% fatty acid free BSA (Roche). During incubations with palmitate serum-free medium was used for MIN6 cells. KRBH buffer contained 115 mmol/L NaCl, 24 mmol/L NaHCO_3,_ 5 mmol/L KCl, 1 mmol/L MgCl_2_, 1 mmol/L CaCl_2_, 0.2% BSA, and 10 mmol/L HEPES. Human pancreatic islets were kindly provided by Professor Olle Korsgren (Department of Radiology, Oncology and Clinical Immunology, Uppsala University Hospital, Uppsala, Sweden), through the Uppsala facility for the isolation of human islets from Scandinavian brain-dead individuals. After isolation, the islets were cultured free-floating in Sterilin dishes in CMRL 1066 medium (ICN Biomedicals, Costa Mesa, CA, USA) containing 5.6 mmol/L glucose, 10% fetal calf serum, and 2 mmol/L L-glutamine for 1–5 days, and then subsequently transferred to the same culture conditions as those used for palmitate exposure of EndoC-βH1 cells. All cells were kept at 37 °C in a humidified atmosphere with 5% CO_2_.

Etomoxir was from Sigma-Aldrich. GPR40 antagonist (GW1100) was from Calbiochem. The PKC inhibitor Bisindolylmaleimide (GF109203X) and the PKD inhibitor CID755673 were from Tocris Bioscience (Bristol, UK).

### Propidium iodide staining and flow cytometry

A total of 10^5^ EndoC-βH1 cells were plated and pre-cultured as described above in 48-well plates for 24–72 h. The cells were then cultured for various time points with or without 1.5 mmol/L palmitate +2% BSA. Cell numbers and cell viability were determined by incubation with 5 µg/mL propidium iodide for 10 min, followed by trypsinization and flow cytometry analysis using a FacsCalibur instrument (BD).

### Hormone secretion to the culture medium or during batch incubation

EndoC-βH1 cells were plated at a density of 150,000 cells/500 µL and grown in 48-well plates for 24 h. Cells were then cultured with or without 1.5 mmol/L palmitate, in the presence/absence of 28 mmol/L glucose for an additional 72 h. For analysis of hormone secretion, cells were pre-incubated for 30 min with 0.5 mmol/L glucose KRBH buffer/0.2% BSA, followed by 0.5 mmol/L/15 mmol/L glucose for 2 h. Islets were similarly exposed to 1.5 mmol/L palmitate, in the presence/absence of 28 mmol/L glucose for 72 h. For analysis of hormone secretion, islets were incubated for 30 min with 2 mmol/L glucose KRBH buffer/0.2% BSA, followed by 20 mmol/L glucose +1.5 mM palmitate for 30 min. Buffers and cell lysates were analyzed for insulin and IAPP contents using an ultrasensitive human insulin ELISA (Mercodia). IAPP concentrations were analyzed using a human Amylin ELISA (Millipore Corporation, Billerica, MA, USA). All experiments were performed in duplicate and repeated at least three times.

Human islets in groups of five were sonicated in 200 µL H_2_O and analyzed in duplicate for insulin and IAPP contents as given above.

### RNA extraction, cDNA synthesis, and semi-quantitative RT-PCR

Total RNA was purified from EndoC-βH1 cells using the Ultraspec RNA reagent (Biotecx, Houston, TX, USA). TaqMan Reverse Transcription Reagents and TaqMan Gene Expression Assays were used for production of cDNA and detection by real-time RT-PCR (Lightcycler 2.0, Roche), respectively. The genes of interest were normalized to an internal control (GAPDH). The primer list and specifications are given in Supplementary Table 1 (available online).

Gene expression of the target genes were quantified by relative quantification using the comparative CT method. Data represent normalized target gene expression expressed as fold change (2^(-ΔΔCt)^*100, where ΔΔCt = ΔCt (target gene_treat_‐GAPDH_treat_) ‐ Δct (target gene_control_‐GAPDH_control_)).

### GPR40 siRNA transfection

Control and *GPR40* siRNA (Santa Cruz Biotechnology, Inc., Dallas, TX, USA) were diluted and mixed with Dharmafect I (Dharmacon) and allowed to incubate for 15 min at room temperature. Cells were then transfected in serum- and antibiotics-free conditions in F12/DMEM medium for 24 h. The transfection medium was then removed, and cells were cultured with or without 1.5 mmol/L palmitate, in the presence/absence of 28 mmol/L glucose for an additional 24 h, prior to RNA extraction and qPCR analysis.

### Immunocytochemistry (ICC)

Cells were transferred to poly-lysine glass slides using a Cytospin 4 instrument (Thermo Scientific). Glass slides were incubated with 4% paraformaldehyde for 5–10 min, permeabilized in 0.1% saponin, and incubated with primary antibodies overnight. Primary antibodies used: Guinea pig anti-insulin, 1:500 (Sigma Aldrich, MO, USA); mouse anti-IAPP, 1:500 (Sigma Aldrich, MO, USA); rabbit anti-glucagon, 1:1000 (Dako, Denmark); rabbit anti-somatostatin, 1:1000 (Sigma Aldrich, MO, USA). Slides were blocked for 30 min using 2% BSA and then incubated with secondary ALEXA Fluor 488 (1:400) and Hoechst for 1.5 h. Slides were mounted using Fluorescent Mounting Medium (Dako S3023).

### Statistical analysis

Comparisons between multiple groups were made by a one-way ANOVA. Student–Newman–Keul’s *post hoc* test was used. Comparisons between control and single treatment groups were done using two-tailed Student’s *t* test. *p* < 0.05 was deemed statistically significant.

## Results

### Long-term exposure of EndoC-βH1 cells to palmitate increases the IAPP/insulin content ratio and the amount of secreted IAPP in response to a high glucose concentration

**Figure 1.**
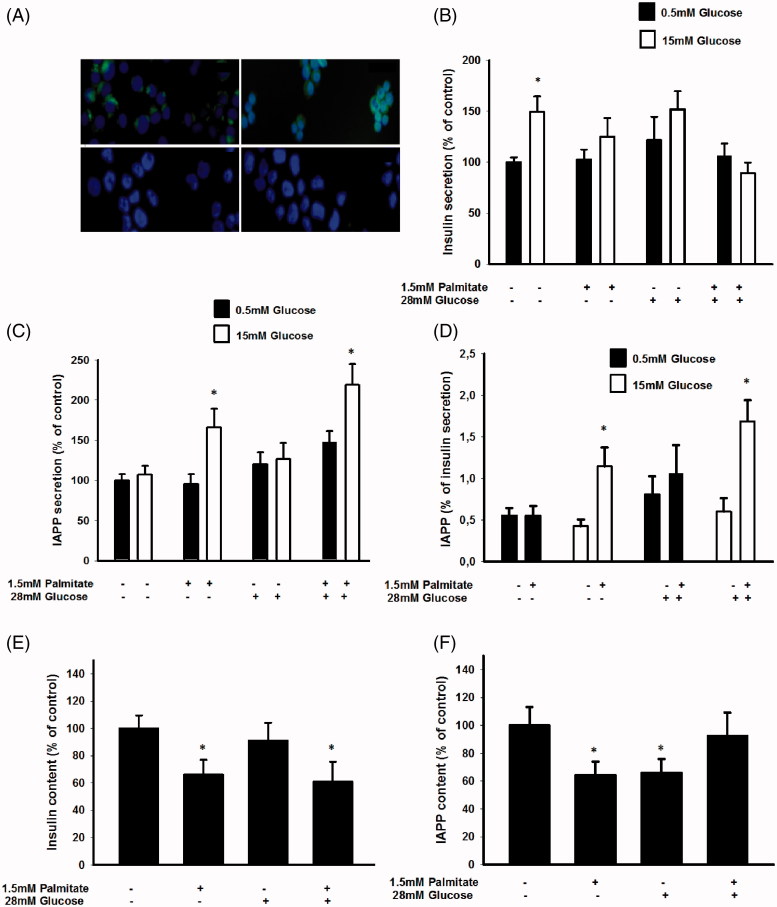
Long-term exposure of EndoC-βH1 cells to palmitate increases glucose-induced IAPP secretion and the IAPP to insulin ratio. (A) EndoC-βH1 cells were immunostained for insulin (upper left), IAPP (upper right), glucagon (lower left), and somatostatin (lower right), as well as with DAPI (blue). The cells were then photographed in a Nikon fluorescence microscope using a 40× lens. (B) EndoC-βH1 cells were cultured for 3 days in 5.6 mmol/L glucose +2% BSA with or without 1.5 mmol/L sodium palmitate and 28 mmol/L glucose. The secretion of insulin was analyzed by incubating the cells at 0.5 or 15 mmol/L glucose in a KRBH buffer for 2 h. (C) Cells cultured for 3 days as given in (B) and then incubated for 2 h in a KRBH buffer were used for IAPP secretion experiments. (D) The percentage of secreted IAPP in relation to insulin was calculated from results obtained in (B) and (C). (E) Cells cultured for 3 days as given in (B) were used for insulin content analysis. (F) Cells cultured for 3 days as given in (B) were used for IAPP content analysis. All results are means of duplicates and are expressed as percent of control. Bars represent means ± SEM for 3–4 independent experiments.

Immunofluorescence analysis confirmed that the human EndoC-βH1 cells exhibit cytoplasmic immunoreactivity for both insulin and IAPP, but not for glucagon or somatostatin (Figure 1(A)).

We next studied the effects of free fatty acids (FFA) *in vitro* on the IAPP/insulin ratio by culturing the EndoC-βH1 cells for 72 h and the murine MIN6 cell line for 24 h with 1.5 mmol/L palmitate, at a fatty acid/BSA molar ratio of 4.95. The palmitate exposure time was set after determining the effects of palmitate on cell viability. An exposure time exceeding 24 h with this concentration of palmitate significantly increased apoptosis in MIN6 cells (results not shown), whereas no effect on cell viability was observed after a 72 h incubation of EndoC-βH1 cells as determined by PI staining and FACS analysis (percentage of dead cells were 18.2%±4.7% in palmitate-treated versus 21.3%±1.3% in control EndoC-βH1 cells, *n* = 3). EndoC-βH1 cells that had been cultured for 3 days at 5.5 mmol/L glucose and without palmitate responded to a high glucose challenge with an increased release of insulin (Figure 1(B)). The EndoC-βH1 cells contained 0.32 ± 0.04 μg insulin/10^6^ cells, secreting 5.65 ± 1.18 ng insulin/h/10^6^ cells at 0.5 mmol/L glucose, and 9.0 ± 0.73 ng insulin/h/10^6^ cells at 15 mmol/L glucose. When cultured in the presence of 1.5 mmol/L palmitate, however, a high glucose concentration no longer stimulated the release of insulin (Figure 1(B)). Also culture at 28 mmol/L glucose abrogated subsequent glucose-stimulated insulin release (GSIS) (Figure 1(B)). IAPP secretion was not stimulated by high glucose after culture at 5.5 mmol/L glucose (Figure 1(C)). The cells contained a mean of 0.18 ± 0.06 ng IAPP/10^6^ cells, and secreted a mean of 16 ± 3 pg IAPP/h/10^6^ cells. However, IAPP release in response to a high glucose concentration was significantly increased in cells cultured with 1.5 mmol/L palmitate both with and without 28 mmol/L glucose (Figure 1(C)). This resulted in a significantly increased IAPP/insulin secretion ratio following a glucose load in cells exposed to palmitate (Figure 1(D)). Palmitate, both by itself and in combination with a high glucose concentration, promoted a decrease in the insulin content (Figure 1(E)). Palmitate culture also reduced the IAPP content of EndoC-βH1 cells (Figure 1(F)). However, cells exposed to palmitate in the presence of 28 mmol/L glucose did not display a reduced IAPP content. These results suggest that palmitate increased the storage and release of IAPP when expressed per insulin.

### Palmitate increases IAPP mRNA and reduces insulin mRNA levels in EndoC-βH1 cells

**Figure 2.**
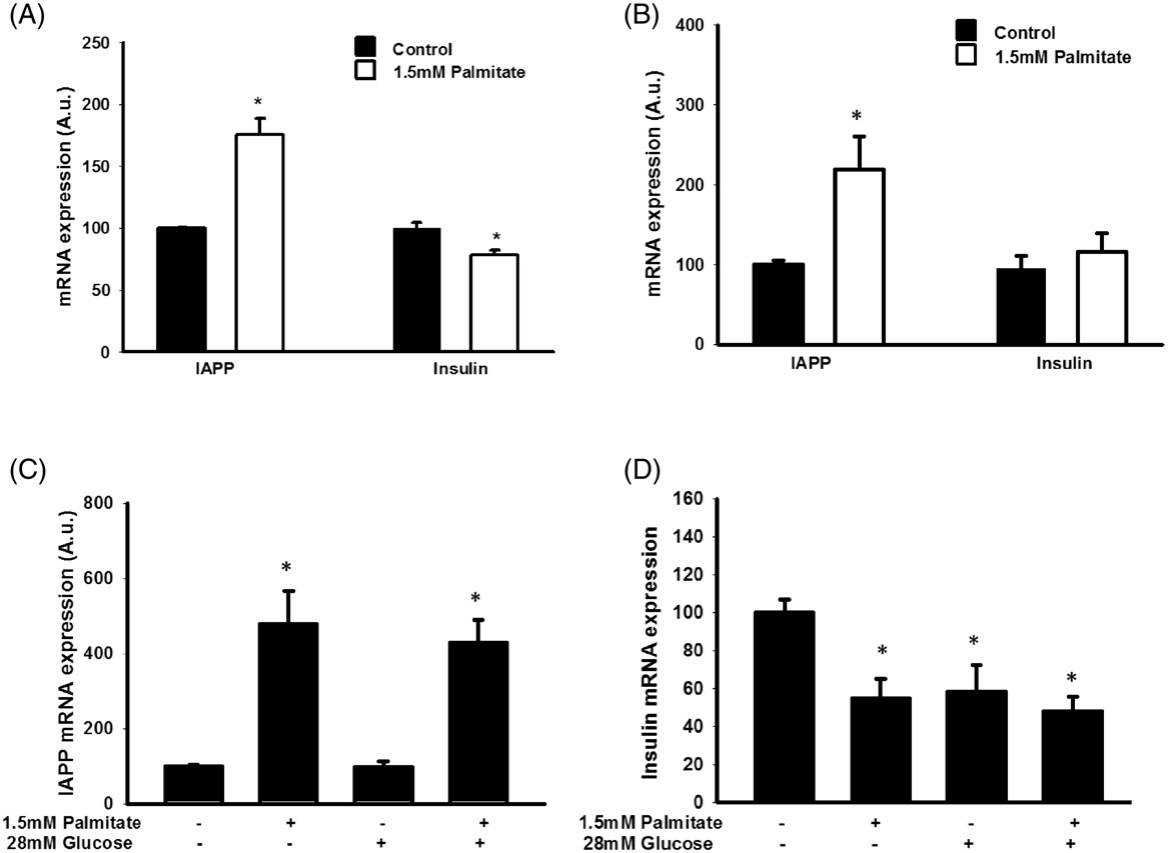
Palmitate increases IAPP mRNA and reduces insulin mRNA. (A) IAPP and insulin mRNA levels were analyzed using real-time RT-PCR. Data represent normalized target gene expression expressed as fold change for IAPP mRNA and insulin mRNA levels of MIN6 cells following palmitate (1.5 mmol/L) exposure for 24 h. (B) EndoC-βH1 cells were cultured for 24 h in the presence of 1.5 mmol/L palmitate and then analyzed for IAPP and insulin mRNA. (C) EndoC-βH1 cells were cultured for 72 h in the presence of 1.5 mmol/L palmitate with and without 28 mmol/L glucose, and then analyzed for IAPP mRNA. (D) EndoC-βH1 cells were cultured for 72 h in the presence of 1.5 mmol/L palmitate with and without 28 mmol/L glucose, and then analyzed for insulin mRNA. Average Ct values; GAPDH: 20.5 ± 0.8, Insulin: 22.4 ± 0.5, IAPP: 26.09 ± 0.7. Bars represent means ± SEM. *p < 0.05 compared with control group. *n* = 4, in duplicate.

To determine whether altered IAPP/insulin protein levels were paralleled by changes in insulin and IAPP mRNA levels, EndoC-βH1 and MIN6 cells were cultured with palmitate and/or a high glucose concentration and then analyzed by real-time RT-PCR. We observed that palmitate increased the expression of IAPP in MIN6 (Figure 2(A)) and in EndoC-βH1 cells (Figure 2(B)) after 24 h. The effects of palmitate on IAPP and insulin mRNA levels were also studied in EndoC-βH1 cells both at 5.5 and 28 mmol/L glucose and after 72 h (Figure 2(C, D)). Also in this case, and at both glucose concentrations, palmitate increased IAPP mRNA. In addition, insulin mRNA was decreased by palmitate and at both glucose concentrations. However, while a high glucose concentration by itself decreased insulin mRNA, no effect was observed on IAPP mRNA levels in response to glucose alone (Figure 2(C, D)).

### The effect of palmitate on IAPP and insulin mRNA levels required GPR40 but not fatty acid oxidation

**Figure 3.**
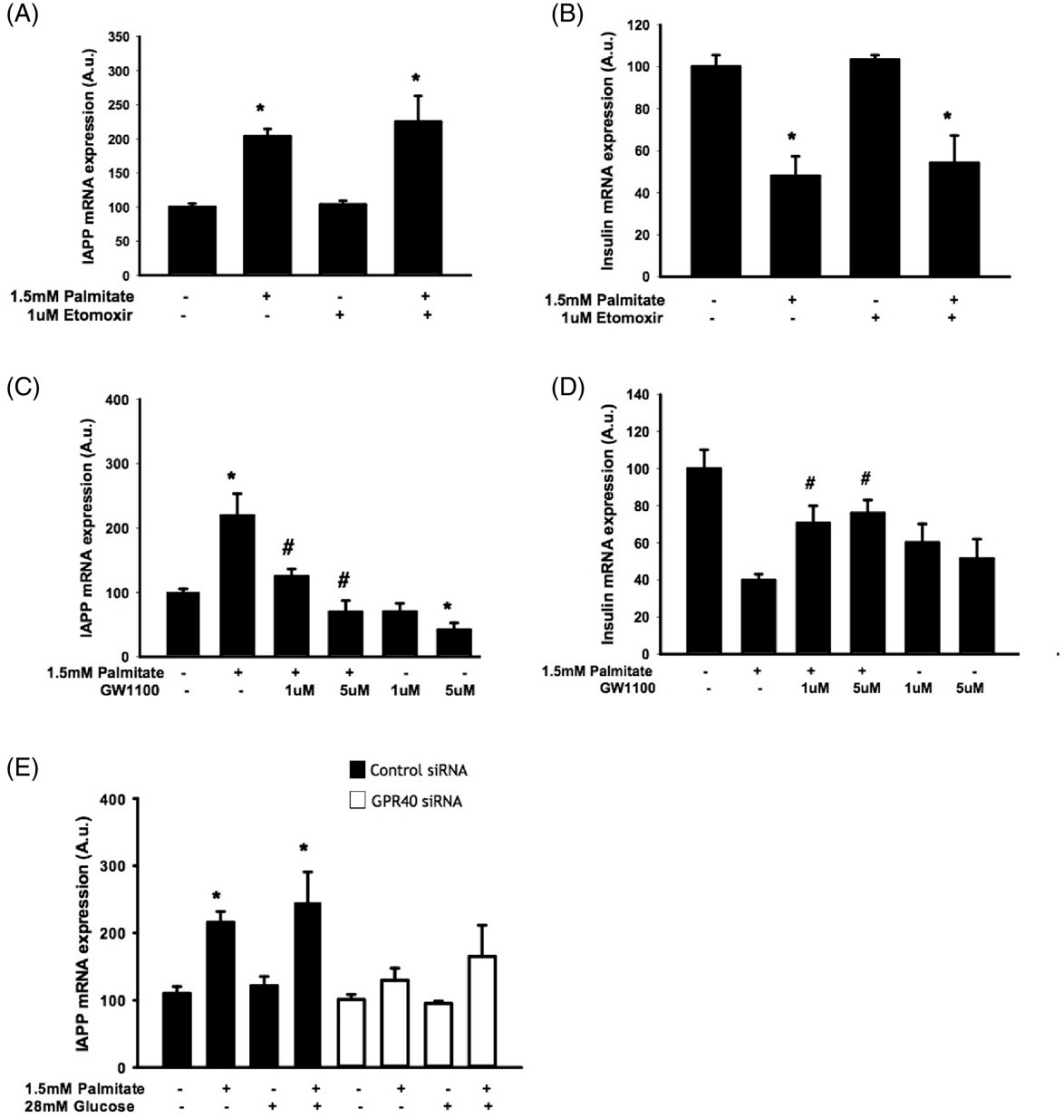
The effect of palmitate on IAPP and insulin mRNA expression is mediated by GPR40 and does not require fatty acid oxidation. EndoC-βH1 cells were cultured for 48 h with or without 1.5 mmol/L palmitate and 1 μmol/L etomoxir and were then analyzed for IAPP (A) and insulin (B) mRNA levels. EndoC-βH1 cells were cultured for 24 h/48 h with or without 1.5 mmol/L palmitate and 1 and 5 μmol/L GW1100 and were then analyzed for IAPP (C) and insulin (D) mRNA levels. Average Ct values; GAPDH: 18.7 ± 0.3, Insulin: 19.4 ± 0.4, IAPP: 21.9 ± 0.2. EndoC-βH1 cells transfected with control/GPR40 siRNA were cultured for 24 h with or without 1.5 mmol/L palmitate and were then analyzed for IAPP (E) mRNA levels. Average Ct values; GAPDH: 18.7 ± 0.9, IAPP: 23.3 ± 1.9. Average Ct values; GAPDH: 24.22 ± 0.2, IAPP: 25.6 ± 0.3. Bars represent means ± SEM. *p < 0.05 compared with control group. #*p* < 0.05 compared with palmitate treated cells. *n* = 3, in duplicate.

To further determine possible involvement of fatty acid oxidation, EndoC-βH1 cells were exposed to palmitate in the presence/absence of etomoxir, an inhibitor of fatty acid oxidation. We observed an intact palmitate-induced increase of *IAPP* mRNA (Figure 3(A)) and a decrease of insulin mRNA (Figure 3(B)) in the presence of etomoxir. We next cultured EndoC-βH1 cells with palmitate in the presence/absence of the GPR40 antagonist GW1100 prior to analysis of *IAPP* and *insulin* gene expression. We demonstrated a GPR40 antagonist-induced dose-dependent inhibition of the palmitate-induced effect on *IAPP* mRNA after 24 h (Figure 3(C)). The effect of palmitate on *insulin* mRNA was not detected after a 24 h exposure (results not shown); instead this effect was observed after 48 h (Figure 3(D)). The palmitate-induced effect on insulin mRNA required GPR40 in 48 h culture experiments as there was a partial antagonizing effect of GW1100 on the palmitate-induced decrease in *insulin* mRNA (Figure 3(D)). In addition, we also observed that *GPR40* siRNA treatment (resulting in 39.5%±13% reduction of receptor expression) prior to palmitate exposure attenuated the subsequent increase in *IAPP* mRNA (Figure 3(E)).

### IAPP-induced signaling may mediate increased IAPP mRNA levels following exposure to palmitate

**Figure 4.**
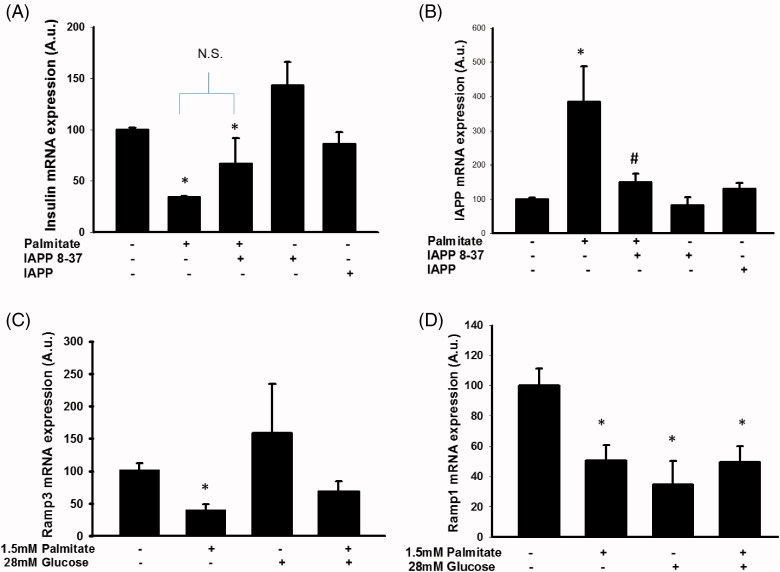
Altered IAPP signaling may underlie enhanced IAPP mRNA expression following exposure to palmitate. EndoC-βH1 cells were cultured for 72 h with or without 1.5 mmol/L palmitate and 6.25 nmol/L IAPP antagonist IAPP 8-37, and were then analyzed for insulin (A) and IAPP (B) mRNA levels. Average Ct values; GAPDH: 18.8 ± 0.2, Insulin: 16.5 ± 0.7, IAPP: 21.6 ± 0.3. Ramp3 (C) and Ramp1 (D) mRNA expression levels were analyzed in EndoC-βH1 cells following culture at 1.5 mmol/L palmitate and/or 28 mmol/L glucose for 72 h. Average Ct values; GAPDH: 19.9 ± 1, Ramp3: 26.8 ± 0.2, Ramp1: 28.5 ± 1.5. Bars represent means ± SEM. *p < 0.05 compared with control group. #*p* < 0.05 compared with palmitate treated cells. *n* = 3–4, in duplicate.

As the palmitate-induced effect on *IAPP* mRNA might precede the effect on *insulin* mRNA, and since IAPP has previously been reported to inhibit insulin secretion (14,15), we investigated a potential role for IAPP signaling in palmitate-mediated regulation of *insulin* mRNA expression. Co-incubation of palmitate with a truncated version of the IAPP peptide (IAPP 8-37) acting as an IAPP antagonist did not significantly alter the effect of palmitate on *insulin* mRNA (Figure 4(A)). Addition of IAPP 1-37 did not reduce *insulin* mRNA expression (Figure 4(A)). However, IAPP 8-37 attenuated the effect of palmitate on *IAPP* mRNA, indicating that increased IAPP signaling mediated the increased expression of *IAPP* mRNA (Figure 4(B)). Furthermore, palmitate also reduced the expression of *Ramp1* and *3* mRNA (Figure 4(C, D)), which further supports an effect of palmitate on the IAPP signaling pathway.

### IAPP-induced signaling may require protein kinase D, but not protein kinase C

**Figure 5.**
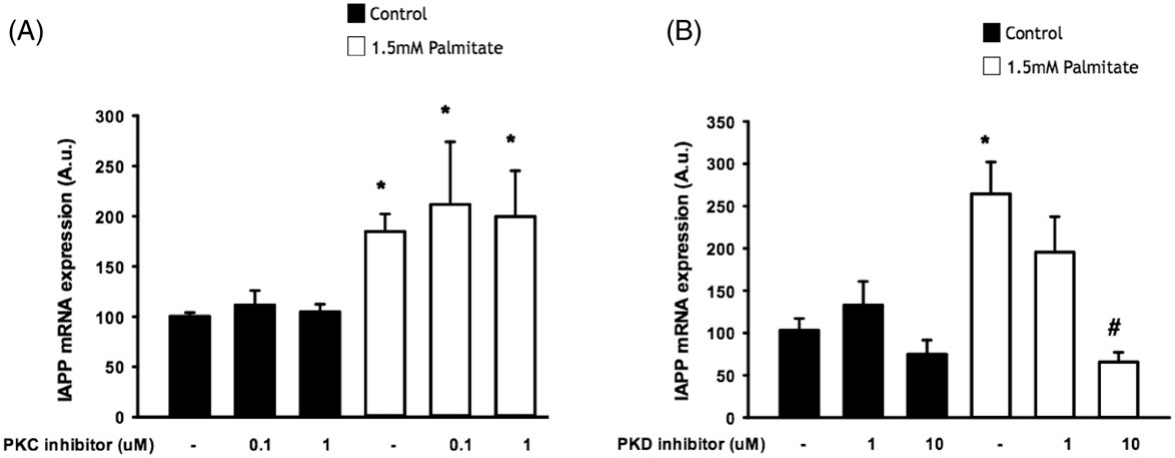
IAPP-induced signaling may require protein kinase D, but not protein kinase C. EndoC-βH1 cells were exposed to 1.5 mmol/L palmitate for 24 h in the presence/absence of different concentrations of a PKC inhibitor (A) and a PKD inhibitor (B), and were then analyzed for IAPP mRNA levels. Bars represent means ± SEM. *Denotes *p* < 0.05 compared with control group. #Denotes *p* < 0.05 compared with palmitate treated cells. *n* = 3, in duplicate.

We next incubated EndoC-βH1 cells with the specific protein kinase C (PKC) inhibitor GF109203X and the PKD inhibitor CID 755673. PKC inhibition failed to alter the effect of palmitate on *IAPP* mRNA expression (Figure 5(A)), while incubation with the PKD inhibitor dose-dependently attenuated the effect of palmitate on *IAPP* mRNA expression, with a significant effect at 10 µmol/L (Figure 5(B)).

### The effect of palmitate on IAPP and insulin mRNA levels is associated with reduced expression of several key transcription factors and increased expression of TXNIP

**Figure 6.**
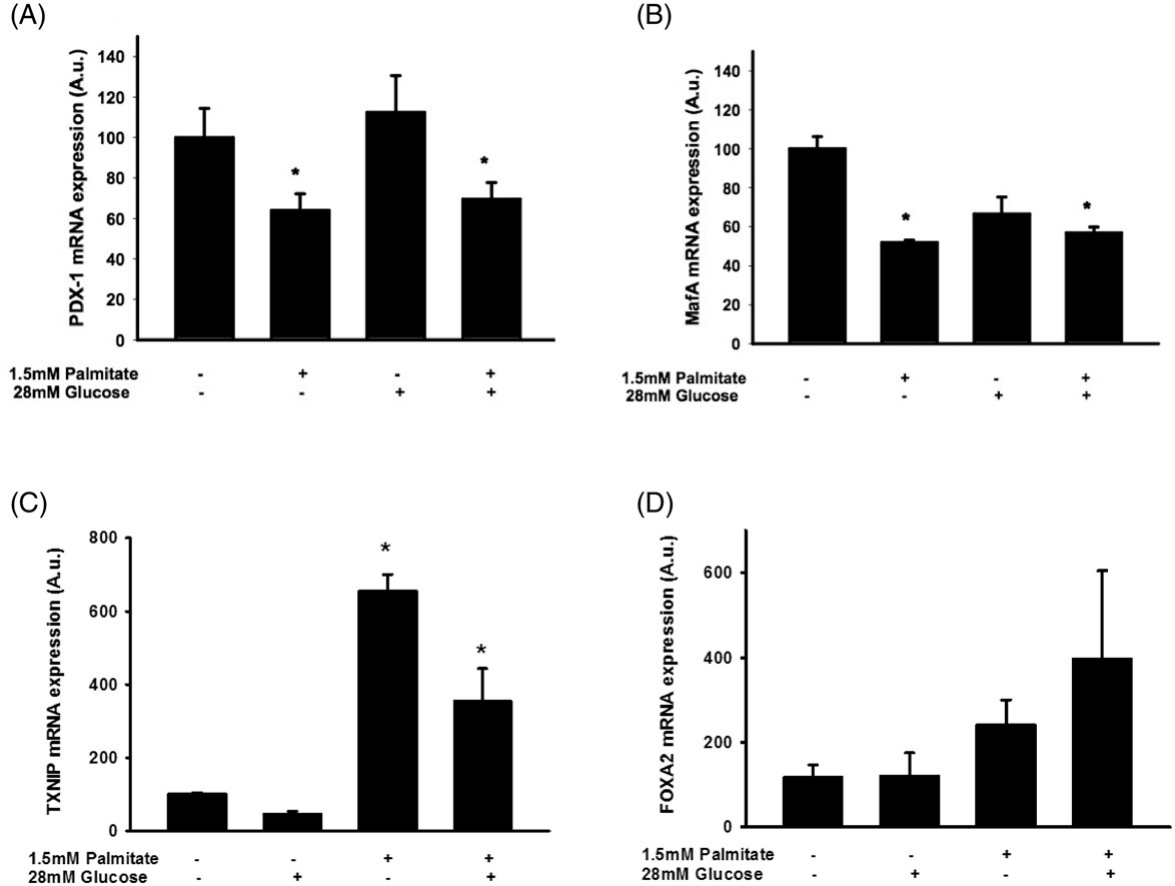
Palmitate reduces the expression of several key transcription factors. EndoC-βH1 cells were cultured for 72 h in the presence of 1.5 mmol/L palmitate with and without 28 mmol/L glucose, and then analyzed for Pdx-1 mRNA (A), MafA mRNA (B), TXNIP mRNA (C), and FoxA2 mRNA (D) by real-time RT-PCR. *n* = 3, in duplicate. Average Ct values; GAPDH: 20.2 ± 0.7, Pdx-1: 29.6 ± 0.8, MafA: 25.1 ± 0.4, TXNIP: 29.0 ± 0.4, FoxA2: 27.1 ± 0.3. Bars represent means ± SEM. *p < 0.05 compared with control group.

To investigate transcriptional events involved in palmitate-induced effects in EndoC-βH1 cells, we used real-time RT-PCR to determine expression levels of key transcription factors. *Pancreatic and duodenal homeobox 1* (*Pdx-1*) gene expression was reduced in EndoC-βH1 cells following exposure to 1.5 mmol/L palmitate in the presence or absence of 28 mmol/L glucose for 72 h (Figure 6(A)). Similarly, *MafA* was reduced in response to long-term exposure to palmitate (Figure 6(B)). On the other hand, mRNA levels of thioredoxin-interacting protein (TXNIP) were potently stimulated by palmitate (Figure 6(C)). There was also a non-significant trend to increased mRNA levels of FoxA2 (Figure 6(D)), a transcription factor that may act cooperatively with TXNIP in augmenting IAPP transcription (30). A high glucose concentration did not affect the mRNA levels of TXNIP and FoxA2 (Figure 6(C, D)).

### Human islet IAPP mRNA levels are increased by both palmitate and a high glucose concentration

**Figure 7.**
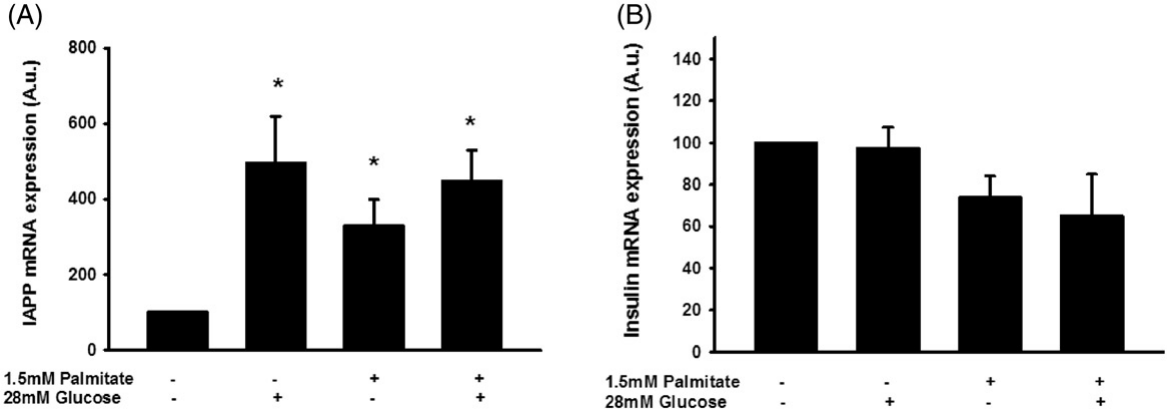
Palmitate and high glucose increase IAPP mRNA, but not insulin mRNA levels. IAPP mRNA levels were analyzed using real-time RT-PCR (A). Data represent normalized target gene expression expressed as fold change for IAPP mRNA levels of human islets following palmitate (1.5 mmol/L) or high glucose (28 mmol/L) exposure for 72 h. (B) Human islets were cultured for 72 h as in (A) and then analyzed for insulin mRNA. Bars represent means ± SEM. *p < 0.05 compared with control group. *n* = 5, in duplicate.

The IAPP mRNA levels of human islets were increased during culture for 3 days in the presence of sodium palmitate (1.5 mmol/L/2% BSA) (Figure 7(A)). The IAPP mRNA levels were similarly increased by a high glucose concentration (28 mmol/L) or a high glucose concentration + palmitate (Figure 7(A)). This was not paralleled by an increased insulin mRNA content; instead there was a trend to lower insulin mRNA levels in response to palmitate or palmitate + a high glucose concentration (Figure 7(B)).

### Human islet IAPP and insulin contents and secretion are differentially affected by palmitate or a high glucose concentration, resulting in an increased IAPP/insulin ratio

**Figure 8.**
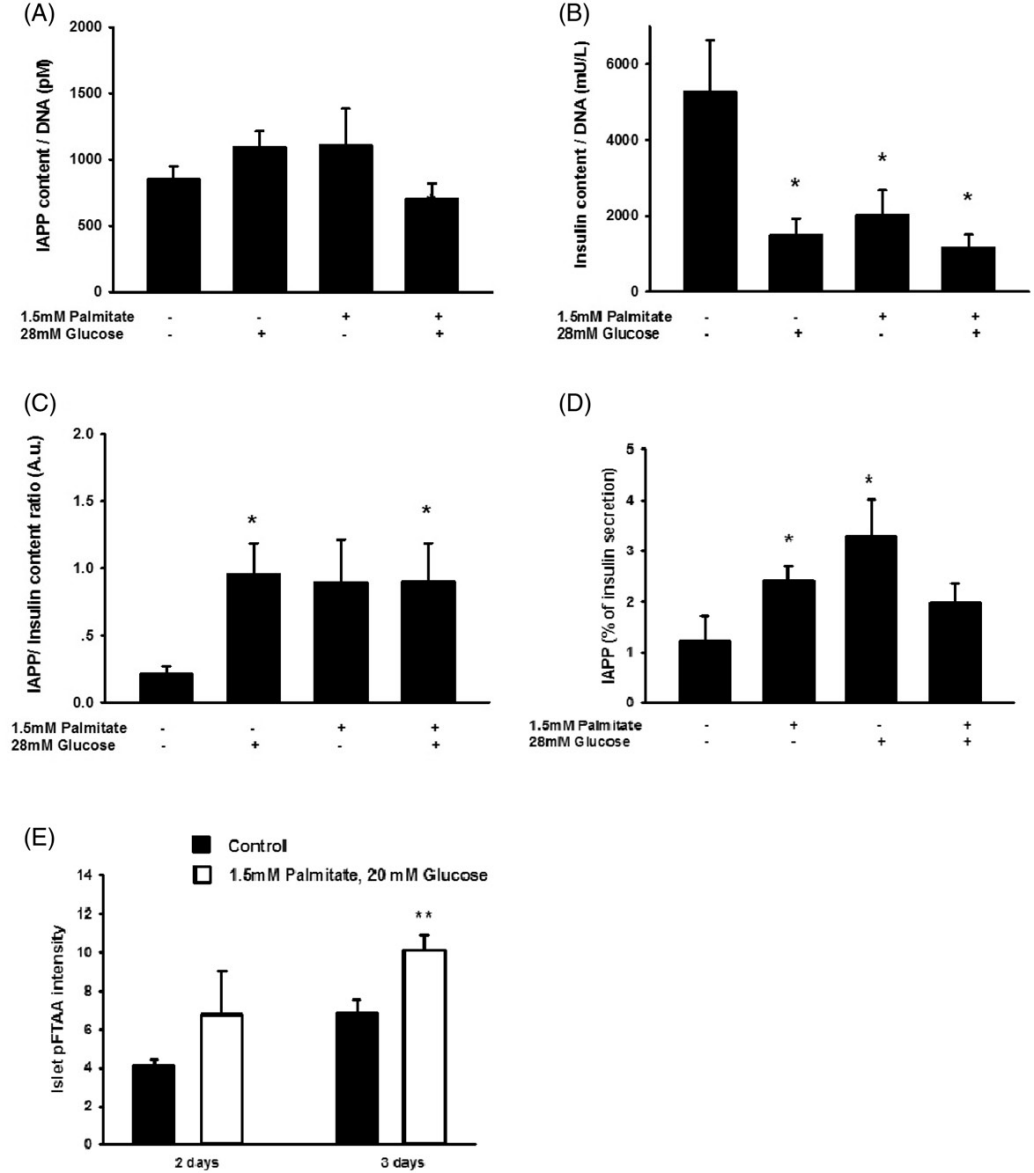
Long-term exposure of human islets to high glucose or high glucose + palmitate. Human islets were cultured for 3 days in 5.6 mmol/L glucose +2% BSA with or without 1.5 mmol/L sodium palmitate and 28 mmol/L glucose, and were used for analysis of IAPP content (A), *n* = 5. (B) Cells cultured for 3 days as given in (A) and used for analysis of insulin content, *n* = 5. (C) The ratio of IAPP in relation to insulin was calculated from results obtained in (A) and (B). (D) The secretion of IAPP and insulin from islets cultured for 3 days as given in (A) was analyzed by incubating the cells at 15 mmol/L glucose in a KRBH buffer for 30 min, and the percentage of secreted IAPP in relation to insulin calculated (D), *n* = 4. (E) Quantification of pFTAA intensity indicate a significant increase in islet amyloid following exposure to high glucose + palmitate for 3 days. All results are means of duplicates. *p < 0.05, **p < 0.01 when comparing versus control using a paired Student's *t* test. Bars represent means ± SEM for 4–7 independent experiments.

We next analyzed human islet IAPP and insulin contents and secretion after culture for 3 days with palmitate/high glucose. It was observed that IAPP contents were not affected by the different culture conditions (Figure 8(A)). Contrastingly, palmitate, a high glucose concentration, and the combination of palmitate and high glucose all depleted insulin contents (Figure 8(B)). This resulted in a marked increase in the ratio between IAPP and insulin contents (Figure 8(C)). In addition, palmitate, or a high glucose concentration, significantly increased the amount of IAPP secreted, while neither exerted a significant effect on insulin secretion, which resulted in an increase in the ratio between IAPP and insulin secreted (Figure 8(D)). Further, quantification of pFTAA intensity indicate a significant increase in islet amyloid following exposure to high glucose + palmitate (Figure 8(E)).

## Discussion

Exogenous palmitate has previously been shown to increase IAPP mRNA levels in murine MIN6 cells (23). This observation was presently reproduced and also extended to human EndoC-βH1 cells, a recently generated cell line that resembles primary human β-cells in regard to stimulus secretion coupling and physiology (24,25). Indeed, in these human β-cells, we demonstrate that addition of exogenous palmitate, present during a long-term culture period, moderately but significantly increases the IAPP/insulin mRNA and protein ratios, and thereby also the ratio of secreted IAPP to insulin. The reduced IAPP content in EndoC-βH1 cells following palmitate exposure observed may result from the high rate of IAPP secretion leading to depletion of IAPP content (26). The increased ratio of secreted IAPP to insulin was only observed after glucose stimulation, and not at a basal glucose concentration. At a non-stimulatory glucose concentration both insulin and IAPP leak out passively or are only constitutively released from the β-cells. It may be that at this condition a higher proportion of non-mature forms of IAPP are released to the exterior of the cells, and as the IAPP ELISA used here does not recognize non-mature forms of the hormone, we could not observe an increased IAPP/insulin ratio in palmitate-treated cells not stimulated with glucose. The effect promoted by palmitate on *insulin* and *IAPP* mRNA levels did not require oxidation of fatty acids, but instead activation of GPR40 was indicated to contribute to the effect of palmitate on IAPP. Interestingly, GPR40, a Gα_q/11_-coupled receptor, has been shown to induce phospholipase C signaling, leading to the intracellular release of IP_3_, diacylglycerol, and Ca^2+^, and a stimulated insulin release (27,28). A short-term GPR40 activation, for example in response to transiently increased levels of circulating FFAs, will certainly help in normalizing blood glucose after a meal, but it may be that long-term GPR40 activation promotes β-cell dysfunction. For example, it has been reported that inhibition of GPR40 leads to protection against palmitate-induced apoptosis *in vitro* (29) and β-cell dysfunction in db/db mice *in vivo* (30). Knockdown or antagonism of GPR40 seems to ameliorate palmitate-induced JNK and p38 activation, and Exendin-4 may protect β-cells, at least in part, via downregulation of GPR40 (31).

The present results, which showed that long-term palmitate-induced GPR40 activation augments the *IAPP/insulin* mRNA ratio, give further support for a deleterious role of GPR40 activation by the saturated fatty acid palmitate, as an increased IAPP/insulin ratio might accelerate the formation of islet amyloid deposits (18), which subsequently stimulate islet macrophages to release pro-inflammatory cytokines (19). Interestingly, inhibition of IAPP signaling using IAPP 8-37 reduced the palmitate-mediated effect on *IAPP* mRNA, suggesting that IAPP-induced autocrine/paracrine effects participate in upregulation of *IAPP* gene expression. This notion is further supported by our finding that palmitate reduced the expression of *Ramp1* and *Ramp3*, which, together with the calcitonin receptor, form IAPP receptors (13). It may be speculated that increased IAPP-induced signaling promotes negative feed-back on *Ramp1/3* expression, but this remains to be experimentally verified. IAPP 8-37 did not significantly alter the effect of palmitate on *insulin* mRNA expression, indicating that palmitate acts on *insulin* gene expression via other signaling pathways.

In this study we also demonstrate that the effect of addition of exogenous palmitate on *IAPP* mRNA appears to require PKD, but not PKC. Differential PKC/PKD dependency can be achieved by G protein α subunits of the Gq family (32). The present lack of PKC-dependency may seem contradictory to an earlier report of PKC-dependency (23). Excluding the fact that human cells were used in this investigation, whereas rodent cells were used in the before-mentioned study (23), the explanation may be the use of different PKC inhibitors. Indeed, Gö-6976 (23) inhibits classical PKC isoforms as well as PKD, while GF109203X used in the present study inhibits both classical and novel PKC isoforms, but does not inhibit PKD, and can thus be used to differentiate between PKD- and PKC-dependent processes (33).

We also demonstrate reduced expression of several transcription factors important for insulin gene expression, which agrees well with the reduced *insulin* mRNA levels observed. The expression of *Pdx-1* and *MafA* were reduced. As the insulin and IAPP genes are equipped with similar control elements in the gene regulatory regions it is likely that some of the transcriptional regulatory mechanisms of these two genes are shared. However, recent studies have, in agreement with the present data, demonstrated dissociation of expression of IAPP and insulin genes. For instance, overexpression of IAPP relative to insulin has been observed in rat models of type 2 diabetes (16), and TXNIP has been shown to inhibit insulin transcription while inducing IAPP transcription (34). The latter finding is supported by the presently observed increase in TXNIP mRNA in palmitate-exposed EndoC-βH1 cells, and a trend to an increased expression of FoxA2, the transcription factor that has been suggested to mediate the increased *IAPP* mRNA transcription (35). Further studies are required to establish the precise roles of TXNIP and FoxA2 in the transcriptional activation of the *IAPP* gene, and whether the effects of palmitate on *IAPP* mRNA expression stem from both transcriptional and post-transcriptional events such as increased stability of *IAPP* mRNA. It is also necessary to elucidate the specific signaling steps induced by palmitate.

Human islets responded to palmitate culture similarly to the human EndoC-βH1 cells, i.e. with increased *IAPP/insulin* mRNA, IAPP/insulin secretion, and IAPP/insulin protein content ratios. This indicates that the results obtained with EndoC-βH1 cells are representative for primary human β-cells. The response of human islets to a high glucose concentration was, however, different from that observed in the EndoC-βH1 cells. The reason for this is unknown, but this difference between EndoC-βH1 cells and primary islets may be due to the contamination of the β-cells in the islets by non-β-cells.

## Conclusions

This study indicates that activation of GPR40 may contribute to the palmitate-induced increase of IAPP/insulin expression and secretion ratios in human insulin-producing cells. Numerous GPR40 agonists have been generated to increase insulin production from β-cells exposed to hyperglycemia *in vivo*, and reports this far provide some proof of concept for GPR40 agonists as a treatment for type 2 diabetes (35). Thus, it may be that the presently observed *in vitro* effects of palmitate are of minor importance to the *in vivo* situation. The possibility remains that extended treatment periods aiming at increasing GPR40 activity could accelerate amyloid deposits and islet inflammation, which might eventually worsen β-cell function. However, the present study also emphasizes the importance of understanding the complexity of GPR40 signal transduction to develop biased agonists achieving improved clinical profile in T2DM.

## Supplementary Material

Supplemental_data_table.pdf
