# Supplementary material for: Addition of exogenous sodium palmitate increases the IAPP/insulin mRNA ratio via GPR40 in human EndoC-βH1 cells
Source: Ups J Med Sci. 2017 Oct 5;122(3):149–59. doi: 10.1080/03009734.2017.1368745 (PMC5649320; doi:10.1080/03009734.2017.1368745)
Supplement: Supplemental_data_table.pdf [file IUPS_A_1368745_SM2362.pdf]

Addition of exogenous Sodium palmitate increases the IAPP/insulin mRNA ratio via GPR40 in human EndoC-βH1 cells.

Camilla Krizhanovskii[*1,2], Rikard G. Fred[1], Marie E. Oskarsson[1], Gunilla T. Westermark[1] and Nils Welsh[1]

Supplemental Table 1 – Primer list.

Name	Sequence	Accession #
GAPDH forward primer	5'-GAGTCAACGGATTTGGTCGT-3'	NM_002046.5
GAPDH reverse primer	5'-GACAAGCTTCCCGTTCTCAG-3'	NM_002046.5
IAPP forward primer	5’-CCTCTACTGCATTCCTCTTG-3’	NM_000415.2
IAPP reverse primer	5’-TCTCATTGTGCTCTCTGTTG-3’	NM_000415.2
Insulin forward primer	5’-TGCTGGTTCAAGGGCTTTAT-3’	NM_000207.2
Insulin reverse primer	5’-GCCTTTGTGAACCAACACCT-3’	NM_000207.2
Pdx-1 forward primer	5'-CCTTGTGCTCGGGTTATGTT-3'	NM_000209.3
Pdx-2reverse primer	5'-ATCATCCCACTGCCAGAAAG-3'	NM_000209.3
Mafa forward primer	5'-GCGGAGAACGGTGATTTCTA -3'	NM_201589.3
Mafa reverse primer	5'- AGGAAAGGGAGGCTGAGAAG-3'	NM_201589.3
GPR40 forward primer	5'-CCACTTCTTCCCACTCTATG-3'	NM_005303.1
GPR40 reverse primer	5'- CCAACCCAAAGACCAGAC-3'	NM_005303.1
TXNIP forward primer	5’-ATCTGAACATCCCTGATACC-3’	NM_006472.5
TXNIP reverse primer	5’-TCAGGGGGCATACATAAAGATAG-3’	NM_006472.5
FoxA2 forward primer	5’-CATGTACGTGTTCATGCC-3	NM_021784.4
FoxA2 reverse primer	5’-CATGTACGTGTTCATGCC-3’	NM_021784.4
